# K^+^ Excretion: The Other Purpose for Puddling Behavior in Japanese *Papilio* Butterflies

**DOI:** 10.1371/journal.pone.0126632

**Published:** 2015-05-08

**Authors:** Takashi A. Inoue, Tetsuo Ito, Hiroshi Hagiya, Tamako Hata, Kiyoshi Asaoka, Fumio Yokohari, Kinuko Niihara

**Affiliations:** 1 Japanese National Institute of Agrobiological Sciences, Ôwashi 1–2, Tsukuba, Ibaraki, 305–8634, Japan; 2 Kibougaoka 34–19, Asahi, Yokohama, Kanagawa, 241–0624, Japan; 3 Department of Natural Science, Faculty of Knowledge Engineering, Tokyo City University, Tamatudzumi, Setagaya, Tokyo, 158–8557, Japan; 4 Division of Biology, Department of Earth System Science, Faculty of Science, Fukuoka University, Shichikuma 8-19-1, Jounan, Fukuoka, Fukuoka, 814–0180, Japan; Onderstepoort Veterinary Institute, SOUTH AFRICA

## Abstract

To elucidate the purpose of butterfly puddling, we measured the amounts of Na^+^, K^+^, Ca^2+^, and Mg^2+^ that were absorbed or excreted during puddling by male Japanese *Papilio* butterflies through a urine test. All of the butterflies that sipped water with a Na^+^ concentration of 13 mM absorbed Na^+^ and excreted K^+^, although certain butterflies that sipped solutions with high concentrations of Na^+^ excreted Na^+^. According to the Na^+^ concentrations observed in naturally occurring water sources, water with a Na^+^ concentration of up to 10 mM appears to be optimal for the health of male Japanese *Papilio* butterflies. The molar ratio of K^+^ to Na^+^ observed in leaves was 43.94 and that observed in flower nectars was 10.93. The Na^+^ amount in 100 g of host plant leaves ranged from 2.11 to 16.40 mg, and the amount in 100 g of flower nectar ranged from 1.24 to 108.21 mg. Differences in host plants did not explain the differences in the frequency of puddling observed for different Japanese *Papilio* species. The amounts of Na^+^, K^+^, Ca^2+^, and Mg^2+^ in the meconium of both male and female butterflies were also measured, and both males and females excreted more K^+^ than the other three ions. Thus, the fluid that was excreted by butterflies at emergence also had a role in the excretion of the excessive K^+^ in their bodies. The quantities of Na^+^ and K^+^ observed in butterfly eggs were approximately 0.50 μg and 4.15 μg, respectively; thus, female butterflies required more K^+^ than male butterflies. Therefore, female butterflies did not puddle to excrete K^+^. In conclusion, the purpose of puddling for male *Papilio* butterflies is not only to absorb Na^+^ to correct deficiencies but also to excrete excessive K^+^.

## Introduction

Puddling (in the narrow sense) is a mostly male behavior observed in Lepidoptera in which individuals sip water from damp ground while urinating. Previous research has shown that the taste of Na^+^ induces puddling in *Papilio glaucus* in North America [1]; *Pieris rapae* in North America [2]; *Papilio helenus* and *P*. *memnon* in Borneo, Malaysia [3]; one of the *Papilio machaon* group of species in North America [4]; and in all the *Papilio* species on the main islands of Japan [5]. The transference of Na^+^ from male butterflies to females during mating has also been observed [6–10]; hence, it has been suggested that the purpose of puddling is to absorb Na^+^ to enhance fertility. This behavior has also been observed in the *Gluphisia* moth [11]. However, only a single study examined whether Na^+^ was actually absorbed into the alimentary tract of Lepidoptera [12]. We photographed many Southeast Asian Danaidae butterflies, particularly of the genera *Tirumala* and *Euploea*, attempting to sip liquid from wet concrete walls or rocks, and some Japanese Lycaenidae butterflies, primarily *Celastrina* spp., sipping water from wet ash. *Papilio* males have also been observed to puddle in wet ash [5]. These butterflies appear to be absorbing substances other than Na^+^ because plant ash contains little Na^+^, and we surmised that *Papilio* butterflies might absorb other substances along with Na^+^. To resolve this issue, the concentrations of substances in both the water that butterflies sip and in their urine need to be measured as in [12]. This type of experiment is difficult to perform with *Papilio* butterflies, and the practical challenges noted in the 1980s seemed insurmountable.

In June 1997, a TV program documenting butterfly behavior was filmed in the greenhouse of “Adachi Park of Living Things, Tokyo.” During this work, we fortuitously observed some male *Papilio* butterflies sipping “Pocari Sweat” (hereafter, PS; this beverage is one of the “ion-supply sports drinks” developed by Otsuka Pharmaceutical Co., LTD., in Japan and is preferred by many nectar-sipping butterflies, including *Papilio* butterflies) that spontaneously changed their behavior to puddling (we did not observe this behavior change in female *Papilio* butterflies). We attempted to obtain butterfly urine and measure the fluctuations of each ion concentration in butterfly alimentary tracts using PS. However, the sensitivity of the analyzing apparatus was too low in the past. Recent advances in analytical apparatus have enabled measurements of very low concentrations of Na^+^, K^+^, Ca^2+^ and Mg^2+^ in small volumes of water using High Performance Liquid Chromatography (HPLC) thus, we began collecting butterfly urine. However, the PS also contained sugars and other organic substances that might result in differences in the concentrations of metal ions in butterfly alimentary tracts. To overcome this obstacle, we sought to identify the type of organic-free mineral water preferred by *Papilio* butterflies. We were most successful in feeding butterflies a solution of 1.0 L of water containing 1.0 g of powdered salt “Yukishio” from Miyakojima, Japan (hereafter, YS01). The concentration of the solution was approximately 13 mM Na^+^.

Almost all butterflies and moths, including *Papilio* species, are dependent on their host plants as food in the larval stage and on flowers in the adult stage, and previous research has shown that plant leaves contain higher amounts of K^+^ than Na^+^ (e.g., http://foodslink.jp/syokuzaihyakka/syun/vegitable/Parsley.htm or http://kta.c.ooco.jp/sp1/sb_seri.html). The authors of [12] measured the amount of each metal ion in the host plant leaves of specific moths; their results supported previous studies. Moreover, the authors of [12] also showed that the urine obtained from the studied moths contained higher amounts of K^+^ compared with moths fed solutions which contained 0 mM or 1 mM K^+^ and assumed that K^+^ excretion is another purpose for puddling in Lepidoptera. In Japan, puddling by *P*. *machaon* in the field is rare compared with that of other Japanese *Papilio* species, although *P*. *machaon* is one of the most common butterfly species in Japan and its taste preferences are the same as those of other *Papilio* species [5]. *P*. *machaon* is the only Apiaceae feeder species among the Japanese *Papilio* species, and this characteristic might affect the need to puddle. Among Japanese Papilionini species, *Parides alcinous* also rarely participates in puddling and is the Papilionini species in Japan that feeds on Aristolochiaceae. Thus, we expanded our work to measure the concentration of metal ions in the leaves of Japanese Papilionini host plant species to determine whether there is a relationship between puddling frequency and metal ion concentration in host plants. The authors of [13] showed that flower nectar contained higher amounts of K^+^ than Na^+^. We also measured the concentration of metal ions in the nectar of flowers that *Papilio* butterflies commonly visit to determine whether there is a relationship between puddling and metal ion concentrations in nectar.

As above, the authors of [12] assumed that K^+^ excretion is another purpose for puddling in Lepidoptera. We hypothesized that butterflies must also excrete K^+^ when they emerge from their pupae because each individual excretes meconium at this time, and the meconium appears to contain K^+^. To investigate this possibility, we measured the amounts of the four metal ions in butterfly meconium. Puddling by females is rare in many butterfly species (except *Appias*, *Limenitis* and certain Polyommatinae butterflies) for reasons that are not completely understood [14]. Female butterflies may be able to obtain Na^+^ from male butterflies as a nuptial gift [6–11]. In addition, because puddling functions to excrete K^+^, we assumed that rare puddling in female butterflies might be a result of K^+^ excretion through their eggs. To investigate this possibility, we collected eggs laid by non-mated laboratory females and measured the amounts of the four metal ions in the eggs.

## Materials and Methods

All the *Papilio* species that occur on the main island of Japan (*P*. *machaon*, *P*. *xuthus*, *P*. *maackii*, *P*. *bianor*, *P*. *helenus*, *P*. *protenor*, *P*. *macilentus* and *P*. *memnon*) were used for the experiments. These butterflies are generally common in Japan and are not protected by law; thus, the butterflies or their larvae were easily obtained from locations where permission for collecting butterflies for experiments was not necessary, such as in our home gardens or in those at our institute.

To collect urine, male butterflies that had primarily emerged in our laboratory were used. Although certain experiments were performed using a portable butterfly cage placed next to puddling sites in the field, in most cases, experiments were performed in the laboratory in the Japanese National Institute of Agrobiological Sciences in Tsukuba, Ibaraki (NIAS). Experiments in the field were try to perform at Yahiko, Niigata (N37:42:35, E138:48:42); Ibusuki, Kagoshima (N31:14:07, E130:35:04) and Aridagawa, Wakayama (N34:03:01, E135:23:42). We also collected one male *P*. *maackii* in Shisô, Hyôgo (N35:00:58, E134:40:44; he was hit-and-run by car and fallen down on the road, still alive) and one male *P*. *helenus* in Yokohama, Kanagawa (N35:20:45, E139:35:26; he flied normally) and used for collecting urines. Emerged butterflies in our laboratory and collected *P*. *maackii* in Shisô and *P*. *helenus* in Yokohama were introduced into rearing cages in the laboratory of NIAS. A polyethylene tube filled with a 50 mM sucrose solution and marked with an artificial flower petal was tethered to the ceiling of the rearing cage. The sucrose solution came up from the tube to the upper part of the petal along a paper string that pierced the artificial petal. In the rearing cages, butterfly health was maintained in good condition, and the butterflies lived almost two weeks. For butterflies that emerged in our laboratory, butterflies at two or three days old were used. When the experiments were performed, each butterfly was individually transferred from the rearing cage to the experimental cage, with the same structure as that of the rearing cage, and the fluid in the polyethylene tube was replaced with a test solution. In this experiment, the butterflies were initially fed YS01 or PS, and according to the results, a solution that contained 5.0 g of powdered salt in 1.0 L of Milli-Q purified water (YS05) was added as a test solution. The contents of Na^+^, K^+^, Ca^2+^, and Mg^2^ are shown in Table 1. The butterflies were removed from the emergence cage or transported from the field and placed on artificial flower petals, and the proboscis was stretched by a needle and attached at the tip to a paper string soaked with test solution. When the butterfly began to puddle, medical urine cups designed for a hospital urine test were placed below the butterfly to collect the urine drops directly. Because the first and second urine drops were typically muddy and colored, it was assumed that these drops contained substances unrelated to puddling. Therefore, we collected urine starting with the first transparent and colorless drop. Urine collection was continued until the butterfly stopped puddling. After this experiment, all butterflies were returned to rearing cages or released to the field from which they were captured. The urine of each individual was transferred into a disposable 15 or 50 mL polyethylene tube (Scientific Specialties Inc., CA, USA, or Becton Dickinson Labware, NY, USA). The concentrations of original solutions which the butterflies were fed were also measured and used to calculate concentration fluctuations of each ion.

**Table 1:** The contents of Na^+^, K^+^, Ca^2+^, and Mg^2+^ in each solution (as packaged).

Materials	Na (mg)	K (mg)	Ca (mg)	Mg (mg)
Powdered salt (1 g) solution	303.0	8.6	6.3	28.1
Pocari Sweat (1 L)	520	227	23	6

To measure the concentrations of metal ions in the leaves of each host plant frequented by Japanese Papilionini butterfly species, the leaves from each host plant species were homogenized in Milli-Q water at a rate of 5 or 10 times the raw leaf weight using Polytron (KINEMATICA, Switzerland). The solution was filtered and transferred into a disposable polyethylene tube. Samples were collected from *Citrus junos* (Rutaceae), *Zanthoxylum ailanthoides* (Rutaceae), *Phellodendron amurense* (Rutaceae), *Orixa japonica* (Rutaceae), *Petroselinum crispum* (Apiaceae), *Oenanthe javanica* (Apiaceae), *Cinnamomum camphora* (Lauraceae) and *Aristolochia debilis* (Aristolochiaceae). All of these samples were collected from the garden or backyard of NIAS (N36:03:12, E140:05:24), and leaves of approximately 10 g for each collected species were examined simultaneously.

To collect flower nectar, the flowers of each plant species that were fed on by Japanese Papilionini butterflies were placed in 50 mL polyethylene tubes with a KIMWIPE tissue (Nippon Paper Crecia Co., Ltd.) and centrifuged for 2 h at 2,380 G in a TOMY EX-125 centrifuge. The KIMWIPEs that had absorbed flower nectar in each polyethylene tube were taken out from the tube, their surfaces were swept with a brush to avoid contamination from pollen and other flower accessories, and were put back in each tube. The ions in the nectar of each species of flower absorbed by the KIMWIPE were dissolved in Milli-Q water at a rate of 5 to 10 times the obtained nectar weight. The obtained each nectar weight (W_nectar_) was then calculated according to W_nectar_ = (weight of KIMWIPE after centrifuge with tube)–(that before centrifuge with tube). Samples were collected from the flowers of *Lycoris radiata* (Amaryllidaceae), *Clerodendrum trichotomum* (Lamiaceae), *Rhododendron macrosepalum* (Ericaceae), *Staphylea bumalda* (Staphyleaceae), *Weigela coraeensis* (Caprifoliaceae), *Lonicera japonica* (Caprifoliaceae), *Citrus unshiu* (Rutaceae), *Lilium auratum* (Liliaceae), *Cayratia japonica* (Vitaceae), *Cirsium japonicum* (Asteraceae), *Aster fastigiatus* (Asteraceae), *Lysimachia clethroides* (Primulaceae), *Robinia pseudoacacia* (Fabaceae) and *Albizia julibrissin* (Fabaceae). *Staphylea bumalda*, *Robinia pseudoacacia*, *Lilium auratum* and *Lysimachia clethroides* were collected from Takao, Hachiôji, Tôkyô (N35:38:05, E139:14:29); *Lycoris radiata*, *Clerodendron trichotomum*, *Cirsium* sp, *Aster fastigiatus*, *Lonicera japonica*, *Citrus sp*, and *Cayratia japonica* were collected from Ôiso, Kanagawa (N35:18:47, E139:17:09); *Weigela coraeensis* was collected from Mizunuma, Kitaibaraki, Ibaraki (N36:51:13, E140:40:04); and *Rhododendron macrosepatum* and *Albizia julibrissin* were collected from the garden or back yard of NIAS.

To collect the meconium, we glued each butterfly pupa to the inner bottom of a 300 mL disposable polystyrene drinking cup using Cemedine Super-X Gold (Cemedine Co., Ltd.). A paper foothold made of a KIMWIPE tissue was attached to the inner sidewall of each cup for the emerged butterfly. After butterfly emergence, the inside of the cup was rinsed with 40 mL of Milli-Q water, and the water, with the pupal case and paper foothold, was transferred to a disposable 50 mL polyethylene tube. All *Papilio* butterfly pupae that could be obtained were used, which included male and female samples. All emerged butterflies were introduced into the rearing cages and were used for other experiments or to obtain the next generation.

To measure the concentrations of the four metal ions in eggs, the eggs laid by non-mated females were crushed in Milli-Q water at a rate of 5 times the obtained egg weight to dissolve the ions in water. The eggs of *P*. *xuthus*, *P*. *maackii*, *P*. *bianor*, *P*. *helenus* and *P*. *protenor* were collected from non-mated females.

The fluid samples were stored at -20°C until analysis. As described in [5], the fluid samples were analyzed with ion chromatography using a PU2080i solvent delivery pump (JASCO, Tokyo, Japan), a JASCO CO-2060 column oven, a CD-5 conductometric detector (Shodex, Kawasaki, Japan), and a JASCO DG-2080-53 online degasser. The separations were achieved on a 4.6-mm ID × 125-mm length fused silica gel column coated with a carboxylic polymer (Shodex IC Y-421) using a guard column (Shodex IC YK-G). The mobile phase consisted of water with 5.0 mM of tartaric acid, 1.0 mM of dipicolinic acid, and 24 mM of boric acid, with a flow rate of 1.0 mL/min. The column oven temperature was 40°C, and the detector temperature was 45°C. The injection volume was 10 μL. Most samples were injected without dilution, but high-concentration samples were diluted 10-fold. All samples were filtered through a 0.45-μm filter (GL Sciences, Tokyo, Japan) before injection. Tartaric acid, dipicolinic acid, and boric acid were analytical grade and were purchased from Wako Pure Chemical Industries (Ôsaka, Japan). Distilled water was HPLC grade and was also purchased from Wako Pure Chemical Industries. The concentration of each ion was determined with calibration curves that were obtained from five concentrations in the range of 5–100 mg/L. The 1.0 g/L solution standards for Na^+^, K^+^, Ca^2+^, and Mg^2+^ were purchased from GL Sciences. The peak areas of the standards were plotted against the concentrations.

The amount of absorbed/excreted ions between the original solution and urine was calculated using the following equation: (O-U) * V, where O is the ion concentration of the original test solution given to the butterfly, U is the ion concentration of the butterfly urine, and V is the urine volume. The amounts of ions in other samples were calculated using the following equation: O * V, where O is the ion concentration in the material solution and V is the volume of the material solution.

## Results

Except for *P*. *machaon* butterflies, most of the prepared butterflies could be used. Almost all of the *P*. *machaon* butterflies rejected attempts to collect urine, and only five butterflies (two on YS01, one on PS, and two on YS05) produced urine. We were able to obtain urine samples from 139 *Papilio* butterflies; three of the butterflies were captured and examined in the field, with one *P*. *machaon* from Yahiko, Niigata and one *Papilio protenor* from Ibusuki, Kagoshima and another *P*. *protenor* from Aridagawa, Wakayama. These butterflies tried to find a puddling site. In addition, as above, a *P*. *maackii* that was picked up in Shisô, Hyôgo, and a *P*. *helenus* that was captured in Yokohama, Kanagawa were taken to the laboratory in NIAS and examined. Differences in butterfly origin or urine collection sites did not appear to affect whether the butterflies puddled. The body sizes of each species are in the order *P*. *machaon* ≈ *P*. *xuthus* ≤ *P*. *macilentus* ≤ *P*. *bianor* ≤ *P*. *maackii* ≈ *P*. *protenor* ≈ *P*. *helenus* ≤ *P*. *memnon*, and in Tables 2–4, the species are arranged in this order. All 44 butterflies that sipped YS01 absorbed Na^+^ and excreted K^+^ (Table 2). The amounts of absorbed Na^+^ were between 5.01 and 2072.5 μg and that of excreted K^+^ were between 9.13 and 3219.26 μg. The correlation coefficient between absorbed Na^+^ and urine volume was 0.44 and between excreted K^+^ and urine volume was 0.43. The molar ratio of absorbed Na^+^ to excreted K^+^ for YS01 was 1.59 ± 0.28 (mean ± SE), and the correlation coefficient between absorbed Na^+^ and excreted K^+^ was 0.93. However, 10 of the 45 butterflies that sipped PS excreted Na^+^ and three absorbed K^+^, whereas the others excreted K^+^ (Table 3). In tests using YS05, 36 out of 50 butterflies excreted Na^+^, and all of the butterflies excreted K^+^ (Table 4). For all three test solutions, no differences were observed in the concentrations of Ca^2+^ and Mg^2+^ between the original solution and urine of all butterflies (Table 2–4). In total, the urine volumes ranged from 0.1 mL to 24 mL, and a specific relationship between urine volume and species was not observed except for *P*. *machaon*. Specific relationship between body size and urine volume, between apparent butterfly origin of the butterfly and urine volume, between apparent butterfly origin and ratio of absorption and excretion, and between puddling duration and absorption and excretion speed were also not observed. The average urine volume from the butterflies that puddled on YS05 was less than that from the butterflies that puddled on the other two test solutions, and urine volume excreted by *P*. *machaon* from all test solutions were also less than those of the other species (Tables 2–4). In our HPLC setup, NH_4_^+^ ions were also detected (http://www.shodex.com/en/dc/07/03/04.html) and observed in certain urine samples; however, neither NH_4_^+^ nor any source substances were detected in the original solutions.

**Table 2:** Absorption and excretion of Na^+^, K^+^, Mg^2+^, and Ca^2+^ in *Papilio* butterflies (YS01).

Species ^a)^	n =	Na^+^ (μg) ^b)^	K^+^ (μg) ^b)^	Ca^2+^ (μg) ^b)^	Mg^2+^ (μg) ^b)^	Urine-V (mL) ^b)^	Na(mol)/K(mol) ^b)^
*P*. *machaon*	2	33.53	-9.13	0.02	1.07	0.12	1.42
		19.27 ± 14.26	-24.56 ± 15.42	-0.12 ± 0.14	0.58 ± 0.49	0.07 ± 0.05	1.18 ± 0.25
		5.01	-39.98	-0.27	0.10	0.02	0.98
*P*. *xuthus*	12	257.46	-33.94	6.69	12.14	6.30	12.86
		153.41 ± 21.07	-205.77 ± 40.13	-10.46 ± 4.08	0.45 ± 1.23	2.92 ± 0.56	2.21 ± 0.98
		19.50	-457.31	-42.08	-4.46	0.25	0.68
*P*. *macilentus*	4	703.78	-37.98	-3.27	0.79	7.00	3.72
		367.77 ± 173.73	-386.98 ± 240.69	-14.74 ± 6.58	-7.82 ± 4.75	3.50 ± 1.74	1.93 ± 0.62
		26.40	-1085.95	-28.97	-17.23	0.50	0.68
*P*. *bianor*	6	454.37	-266.28	-3.78	4.00	12.00	1.68
		247.32 ± 51.99	-394.08 ± 30.12	-35.14 ± 10.43	-1.02 ± 1.89	7.57 ± 1.27	1.04 ± 0.17
		105.82	-461.03	-81.38	-6.86	5.00	0.48
*P*. *maackii*	10	2072.54	-165.32	-4.78	6.95	24.00	2.97
		537.89 ± 180.83	-748.30 ± 281.41	-54.05 ± 14.71	-0.23 ± 2.55	11.20 ± 1.98	1.34 ± 0.21
		104.61	-3219.26	-144.39	-19.01	2.00	0.57
*P*. *protenor*	5	811.23	-22.11	6.55	5.83	7.00	1.53
		316.55 ± 133.99	-520.90 ± 214.59	-14.25 ± 9.34	0.16 ± 1.61	3.91 ± 1.34	1.09 ± 0.17
		10.54	-1134.35	-47.62	-3.50	0.05	0.60
*P*. *helenus*	4	1714.76	-30.94	-2.63	0.00	15.00	2.09
		624.90 ± 377.69	-622.48 ± 398.29	-43.48 ± 15.86	-12.38 ± 14.22	5.38 ± 3.26	1.86 ± 0.10
		38.19	-1785.71	-73.79	-54.90	0.50	1.63
*P*. *memnon*	1	-	-	-	-	-	-
		457.57	-787.90	-47.93	4.12	6.00	0.98
		-	-	-	-	-	-
Total	44	2072.54	-9.13	6.69	12.14	24.00	12.86
		335.30 ± 60.50	-449.91 ± 83.36	-27.94 ± 4.91	-1.77 ± 1.52	5.76 ± 9.25	1.59 ± 0.28
		5.01	-3219.26	-144.39	-54.90	1.60	0.48

^a)^ Species are arranged according to their approximate body size from top to bottom.

^b)^ In this column, the species’ maximum value, mean ± SD, and minimum value are shown from top to bottom.

**Table 3:** Absorption and excretion of Na^+^, K^+^, Mg^2+^, and Ca^2+^ in *Papilio* butterflies (PS).

Species ^a)^	n =	Na^+^ (μg) ^b)^	K^+^ (μg) ^b)^	Ca^2+^ (μg) ^b)^	Mg^2+^ (μg) ^b)^	Urine-V (mL) ^b)^	++/-+/−− ^c)^
*P*. *machaon*	1	-	-	-	-	-	0
		3.41	-1.16	-12.28	-3.38	1.00	0
		-	-	-	-	-	0
*P*. *xuthus*	8	477.46	-1.94	-23.96	66.24	7.00	0
		158.03 ± 88.66	-500.11 ± 103.60	-74.65 ± 19.07	-21.06 ± 15.09	4.06 ± 0.64	0
		-267.98	-964.75	-196.19	-81.15	2.00	2
*P*. *macilentus*	5	810.00	-195.69	-17.83	-6.91	11.00	0
		117.98 ± 208.17	-648.26 ± 258.98	-113.89 ± 56.73	-39.96 ± 26.39	4.70 ± 2.19	0
		-440.11	-1449.60	-331.69	-144.78	100.00	3
*P*. *bianor*	9	4005.00	1650.00	238.38	48.90	15.00	2
		1027.16 ± 446.67	-58.68 ± 248.32	21.10 ± 50.22	0.84 ± 10.49	7.61 ± 1.60	0
		-64.29	-1144.77	-165.86	-44.14	0.10	1
*P*. *maackii*	9	1850.44	120.88	246.05	93.77	14.00	1
		243.35±535.81	-408.60±142.54	29.40±49.44	14.63±14.14	16.67±1.15	0
		-3966.22	-1266.14	-276.58	0.00	3.00	1
*P*. *protenor*	8	996.40	-171.71	118.16	23.78	14.00	0
		360.70 ± 169.88	-935.78 ± 227.24	-149.79 ± 65.48	-32.17 ± 14.63	6.88 ± 1.61	0
		-544.25	-2230.43	-451.58	-96.25	2.00	1
*P*. *helenus*	4	847.90	-0.67	321.88	99.31	14.00	0
		100.75 ± 366.06	-587.30 ± 283.52	0.97 ± 90.99	4.50 ± 32.01	8.60 ± 2.31	0
		-765.42	-1224.16	-125.66	-78.45	3.00	2
*P*. *memnon*	1	-	-	-	-	-	0
		457.57	-787.9	-47.93	4.12	6.00	0
		-	-	-	-	-	0
Total	45	4005.00	1650.00	321.88	99.31	15.00	3
		362.72 ± 152.23	-476.05 ± 88.18	-40.43 ± 22.65	-9.44 ± 6.85	6.15 ± 0.62	0
		-3966.22	-2230.43	-451.58	-144.78	0.10	10

^a)^ Species are arranged according to their approximate body size from top to bottom.

^b)^ In this column, the species’ maximum value, mean ± SD, and minimum value are shown from top to bottom.

^c)^ Column from top to bottom: the numbers of individuals that absorbed both Na and K, excreted Na and absorbed K, excreted both Na and K.

**Table 4:** Absorption and excretion of Na^+^, K^+^, Mg^2+^, and Ca^2+^ in *Papilio* butterflies (YS05).

Species ^a)^	n =	Na^+^ (μg) ^b)^	K^+^ (μg) ^b)^	Ca^2+^ (μg) ^b)^	Mg^2+^ (μg) ^b)^	Urine-V (mL) ^b)^	++/-+/−− ^c)^
*P*. *machaon*	2	114.00	-55.95	0.71	7.00	0.50	0
		-147.56 ± 261.56	-95.73 ± 39.77	-16.66 ± 17.37	-17.07 ± 24.07	0.50 ± 0.00	0
		-409.11	-135.50	-34.02	-41.14	0.50	1
*P*. *xuthus*	7	-46.9	-70.99	-14.98	-7.50	3.00	0
		-362.84 ± 78.98	-178.66 ± 29.12	-22.10 ± 1.95	-37.47 ± 7.40	1.81 ± 0.29	0
		-609.41	-253.39	-28.59	-57.31	0.70	7
*P*. *macilentus*	5	7.15	-169.89	15.81	-8.14	5.00	0
		-291.16 ± 97.53	-339.89 ± 118.30	-17.24 ± 9.40	-36.21 ± 12.03	2.40 ± 0.73	0
		-591.43	-805.31	-35.69	-77.34	1.00	4
*P*. *bianor*	13	871.56	-36.87	-5.99	56.74	11.00	0
		-291.80 ± 191.11	-247.62 ± 45.29	-25.97 ± 5.79	-40.58 ± 15.55	3.54 ± 0.89	0
		-1618.31	-497.11	-64.41	-151.10	0.50	8
*P*. *maackii*	6	-271.40	-85.53	00–3.37	-13.00	8.00	0
		-553.34 ± 76.09	-211.22 ± 40.56	-27.24 ± 5.77	-54.14 ± 8.50	4.33 ± 0.95	0
		-794.18	-349.93	-47.00	-68.90	1.00	6
*P*. *protenor*	10	299.39	-21.95	-3.83	6.37	11.00	0
		-217.39 ± 144.69	-592.37 ± 94.76	-28.69 ± 5.89	-47.86 ± 12.59	4.85 ± 1.01	0
		-788.95	-990.74	-56.26	-117.71	1.00	5
*P*. *helenus*	3	115.04	-75.47	-5.77	-13.31	2.00	0
		48.01 ± 58.38	-211.86 ± 89.07	-9.67 ± 2.88	-20.48 ± 3.59	1.17 ± 0.44	0
		-68.30	-379.29	-15.28	-24.53	0.50	1
*P*. *memnon*	4	-8.88	-73.60	-3.53	-6.06	2.00	0
		-41.73 ± 12.05	-123.29 ± 23.18	-12.62 7.15	-13.15 3.74	1.00 0.35	0
		-66.47	-175.83	-33.98	-22.99	0.50	4
Total	50	871.56	-21.95	15.81	56.74	11.00	0
		-272.02 ± 62.23	-293.60 ± 33.98	-22.83 ± 2.42	-38.45 ± 5.26	3.07 ± 0.38	0
		-1618.31	-990.74	-64.41	-151.10	0.50	36

^a)^ Species are arranged according to their approximate body size from top to bottom.

^b)^ In this column, the species’ maximum value, mean ± SD, and minimum value are shown from top to bottom.

^c)^ Column from top to bottom: the number of individuals that absorbed both Na and K, excreted Na and absorbed K, and excreted both Na and K.

The amounts of Na^+^ in the host plant leaves were between 2.11 mg and 16.40 mg per 100 g of leaves, and the amounts of K^+^ in the host plant leaves were between 361.1 mg and 815.5 mg per 100 g of leaves. The molar ratio of K^+^ to Na^+^ in the leaves was between 19.5 and 163.25 and the average was 43.94 (Table 5). In contrast, the amount of Na^+^ in flower nectar was between 1.24 mg and 108.21 mg per 100 g of nectar, and the amount of K^+^ in the nectar was between 2.77 mg and 1053.85 mg per 100 g of nectar. The molar ratio of K^+^ to Na^+^ in the nectar was between 0.02 and 166.74, and the average was 10.93 (Table 6). The Na^+^ concentrations in 7 of the 14 plant species studied were greater than that in YS01, and the concentrations in 3 plants were greater than that of PS.

**Table 5:** Amounts of Na^+^, K^+^, Mg^2+^, and Ca^2+^ in 100 g of Japanese Papilionini host plant leaves.

Species	n =	Na^+^ (μg)	K^+^ (μg)	Ca^2+^ (μg)	Mg^2+^ (μg)	K(mol)/Na(mol)
*Citrus junos*	1	8.70	580.49	189.47	64.90	39.36
*Zanthoxylum ailanthoides*	1	12.91	457.23	253.47	26.08	20.88
*Phellodendron amurense*	1	2.11	584.43	234.55	64.58	163.25
*Orixa japonica*	1	3.77	737.86	192.24	75.22	115.42
*Petroselinum crispum*	1	16.40	815.52	88.99	26.89	29.33
*Oenanthe javanica*	1	3.29	495.52	14.74	11.92	88.77
*Cinnamomum camphora*	1	10.920	361.17	657.11	139.47	19.50
*Aristolochia debilis*	1	4.35	621.13	332.05	45.41	84.14
Total						43.94

**Table 6:** Amounts of Na^+^, K^+^, Mg^2+^, and Ca^2+^ in 100 g flower nectar used by Japanese Papilioninae species.

Species	n =	Na^+^ (μg) ^b)^	K^+^ (μg) ^b)^	Ca^2+^ (μg) ^b)^	Mg^2+^ (μg) ^b)^	K(mol)/Na(mol) ^b)^
*Lycoris radiata*	6	96.16	31.32	74.91	28.45	0.30
		74.83 ± 5.96	23.72 ± 2.15	47.31 ± 6.03	20.72 ± 1.81	0.20 ± 0.03
		55.91	17.87	33.13	15.53	0.13
*Cl*. *trichotomum*	2	77.21	120.02	133.43	38.56	2.64
		72.15 ± 5.06	72.91 ± 47.11	100.32 ± 33.12	31.62 ± 6.94	0.57 ± 0.34
		67.1	25.81	67.2	24.67	0.65
*Rh*. *macrosepatum*	8	108.21	67.41	268.77	71.51	3.94
		44.27 ± 12.14	22.67 ± 8.42	118.63 ± 33.38	33.56 ± 9.03	1.13 ± 0.61
		5.51	2.77	14.59	5.44	0.02
*Staphylea bumalda*	2	5.07	51.5	22.85	25.11	24.5
		3.15 ± 1.91	40.94 ± 10.57	22.52 ± 0.34	21.76 ± 3.35	14.02 ± 10.48
		1.24	30.37	22.18	18.41	3.53
*Weigela coraeensis*	2	6.75	212.48	2.65	6.84	57.92
		4.16 ± 2.59	183.52 ± 28.96	2.27 ± 0.38	5.46 ± 1.38	38.24 ± 19.68
		1.57	154.56	1.89	4.08	18.57
*Cirsium* sp	2	12.53	210.69	38.62	14.19	12.12
		11.39 ± 1.14	174.85 ± 35.84	38.28 ± 0.34	12.52 ± 1.67	9.33 ± 2.79
		10.25	139	37.95	10.85	6.54
*Aster fastigiatus*		68.15	399.78	28.57	14.37	166.74
		34.78 ± 33.37	228.95 ± 170.83	28.28 ± 0.29	11.36 ± 3.01	83.62 ± 83.12
		1.41	58.12	27.99	8.35	0.50
*Lonicera japonica*	2	14.63	55.49	9.81	5.07	2.79
		12.81 ± 1.82	53.75 ± 1.73	8.33 ± 1.48	4.37 ± 0.70	2.51 ± 0.28
		10.99	52.02	6.86	3.67	2.24
*Citrus* sp	2	25.58	139.24	18.9	8.94	3.26
		25.38 ± 0.21	111.17 ± 28.07	17.66 ± 1.25	7.53 ± 1.40	2.59 ± 0.67
		25.17	88.11	16.41	6.13	1.92
*Lilium auratum*	2	37.79	99.29	16.62	5.1	2.19
		32.26 ± 0.34	86.53 ± 12.77	16.45 ± 0.17	4.99 ± 0.11	1.67 ± 0.52
		26.73	73.76	16.28	4.88	1.15
*Ly*. *clethroides*	2	5.21	176.26	25.5	10.61	28.32
		3.83 ± 1.38	147.00 ± 29.26	18.82 ± 6.68	8.08 ± 2.53	24.13 ± 4.19
		2.45	117.74	12.14	5.55	19.94
*Albizia julibrissin*	2	98.14	1053.85	496.33	109.44	12.17
		74.51 ± 23.63	1051.73 ± 2.12	433.85 ± 82.48	91.73 ± 17.71	9.25 ± 2.92
		50.88	1049.6	331.36	74.02	6.3
*Cayratia japonica*	2	41.06	258.51	131.55	32.38	3.71
		37.50 ± 3.55	193.76 ± 64.75	107.29 ± 24.25	25.55 ± 6.83	2.98 ± 0.74
		33.95	129.01	83.04	18.72	2.24
*Ro*. *pseudoacacia*	2	3.38	83.38	27.05	6.69	14.54
		2.77 ± 0.61	64.88 ± 18.50	21.41 ± 5.64	5.26 ± 1.44	13.58 ± 0.96
		2.17	46.38	15.77	3.82	12.63
Total		108.21	1053			166.74
		37.7	135.36			10.93 ± 4.59
		1.24	2.77			0.02

^b)^ In this column, the species’ maximum value, mean ± SD, and minimum value are shown from top to bottom.

All of the male and female butterflies excreted large amounts of K^+^ in the meconium compared with the other three ions. Significant differences were not observed between males and females (Tables 7–8). Certain samples contained NH_4_^+^ ions, which frequently prevented the detection of the Na^+^ peak. In the analysis of meconium, the Na^+^ peak on the HPLC charts was generally small, whereas NH_4_^+^ amounts were relatively large; in certain samples, especially those from females, we could not clearly detect the Na^+^ peak on the HPLC chart. In such cases, we treated the Na^+^ amount as 0. A specific relationship was not observed among body size or butterfly species for any of the ions researched in this experiment.

**Table 7:** Amounts of the four metal ions in the meconium of Japanese male *Papilio* butterflies.

Species ^a)^	n =	Na^+^ (μg) ^b)^	K^+^ (μg) ^b)^	Ca^2+^ (μg) ^b)^	Mg^2+^ (μg) ^b)^
*P*. *machaon*	6	285.12	1893.77	130.34	67.77
		191.71 ± 24.04	1332.86 ± 12.50	77.46 ± 12.50	35.50 ± 6.89
		115.34	918.39	42.86	18.46
*P*. *xuthus*	6	66.62	1314.91	128.94	77.10
		41.40 ± 8.15	888.43 ± 106.88	68.28 ± 14.90	31.27 ± 10.95
		18.80	624.02	35.58	2.66
*P*. *macilentus*	7	19.02	1460.25	185.01	126.35
		3.62 ± 2.63	1112.31 ± 110.68	151.99 ± 7.67	74.66 ± 10.81
		0.00	739.74	134.68	47.46
*P*. *bianor*	6	168.04	1652.61	102.39	73.89
		60.19 ± 27.47	1275.04 ± 92.08	78.05 ± 8.38	50.16 ± 8.24
		0.00	1020.92	44.34	26.33
*P*. *maackii*	6	112.08	2835.49	151.39	119.66
		41.48 ± 19.95	2062.38 ± 322.70	87.68 ± 14.90	79.25 ± 14.98
		0.00	983.70	49.49	11.52
*P*. *protenor*	5	138.20	3032.19	134.38	217.49
		69.29 ± 26.26	2210.36 ± 211.44	92.02 ± 14.96	156.89 ± 21.80
		0.00	1579.85	38.36	77.70
*P*. *helenus*	5	101.1	3319.35	70.65	151.72
		41.58 ± 16.50	2254.62 ± 320.46	58.58 ± 3.73	90.00 ± 26.03
		0.00	1501.84	47.79	0.00
*P*. *memnon*	10	287.50	3694.48	171.06	203.75
		82.29 ± 29.48	2648.65 ± 240.39	75.70 ± 1.29	109.49 ± 19.56
		0.00	1600.40	12.06	54.22
Total	51	287.50	3694.48	185.01	217.49
		66.89 ± 10.61	1772.31 ± 114.04	87.21 ± 5.97	80.50 ± 7.70
		0.00	624.02	12.06	0.00

^a)^ Species are arranged according to their approximate body size from top to bottom.

^b)^ In this column, the species’ maximum value, mean ± SD, and minimum value are shown from top to bottom.

**Table 8:** Amounts of the four metal ions in the meconium of Japanese female *Papilio* butterflies.

Species ^a)^	n =	Na^+^ (μg) ^b)^	K^+^ (μg) ^b)^	Ca^2+^ (μg) ^b)^	Mg^2+^ (μg) ^b)^
*P*. *machaon*	1	-	-	-	-
		98.77	1665.06	88.2	55.68
		-	-	-	-
*P*. *xuthus*	8	203.21	1088.92	256.55	107.81
		119.82 ± 25.31	686.35 ± 115.72	107.19 ± 24.22	49.20 ± 12.68
		0.00	256.80	42.11	4.08
*P*. *macilentus*	9	144.26	1251.61	64.75	165.59
		100.03 ± 6.46	837.53 ± 107.25	39.06 ± 5.21	70.67 ± 15.90
		75.60	339.74	20.45	17.59
*P*. *bianor*	5	398.00	1072.60	88.82	142.92
		125.03 ± 71.67	734.26 ± 109.84	60.45 ± 10.93	69.53 ± 15.40
		10.33	310.97	23.17	44.77
*P*. *maackii*	3	33.82	3994.55	144.51	141.70
		16.66 ± 9.77	2356.71 ± 896.73	78.16 ± 36.99	99.59 ± 21.52
		0.00	904.96	16.66	70.81
*P*. *protenor*	10	202.86	2838.18	214.02	280.00
		88.46 ± 25.77	1994.74 ± 145.54	91.14 ± 24.44	156.20 ± 30.41
		0.00	1341.77	0.00	4.40
*P*. *helenus*	4	171.50	1227.81	146.42	174.65
		80.49 ± 46.67	954.94 ± 107.96	53.66 ± 31.11	97.51 ± 42.00
		0.00	719.99	17.20	11.36
*P*. *memnon*	8	0.00	5981.36	1290.82	314.61
		0.00 ± 0.00	2754.34 ± 515.23	649.43 ± 189.47	199.77 ± 29.85
		0.00	970.70	0.00	50.35
Total	48	398.15	5981.36	1290.82	314.61
		79.99 ± 22.84	1468.80 ± 152.70	167.68 ± 43.09	109.21 ± 11.92
		0.00	256.80	0.00	4.08

^a)^ Species are arranged according to their approximate body size from top to bottom.

^b)^ In this column, the species’ maximum value, mean ± SD, and minimum value are shown from top to bottom.

The proportions of each metal ion in the eggs of the species examined were similar, with the eggs of all species containing more K^+^ than the other three ions. The egg size of each species were in the order *P*. *xuthus* < *P*. *bianor* = *P*. *maackii* < *P*. *helenus* ≤ *P*. *protenor*, and in the Table 9, the species are arranged in this order. Generally, the amounts of each metal ion in the eggs corresponded to the volume of the eggs (Table 9).

**Table 9:** Amounts of the four metal ions in the eggs of five Japanese *Papilio* species.

Species ^d)^	Na^+^ (μg)	K^+^ (μg)	Ca^2+^ (μg)	Mg^2+^ (μg)
*P*. *xuthus*	0.37	2.59	0.12	0.20
*P*. *bianor*	0.46	3.14	0.21	0.28
*P*. *maackii*	0.22	3.65	0.25	0.36
*P*. *protenor*	0.68	4.05	0.29	0.29
*P*. *helenus*	0.64	5.77	0.26	0.37

^d)^ Species are arranged according to their approximate egg size from top to bottom.

## Discussion

The difference in the average amount of Na^+^ absorbed between a previous study [12] and the present study using YS01 was likely caused by a difference in body size between *Papilio* butterflies and the *Gluphisia* moth. As shown in Table 1, in the PS, the concentration of Na^+^ was slightly higher than in the YS01. For a number of butterflies, excreted Na^+^ was positively correlated with the Na^+^ concentration in the original solution, which suggested that high Na^+^ concentrations might affect the alimentary tract of butterflies. This phenomenon was not observed in [12], in which 1 mM Na^+^ solutions only were used. In our previous study, the concentrations of Na^+^ in water that *Papilio* butterflies puddled in the field were up to 6.0 mM [5]. Therefore, the alimentary tract of butterflies was assumed to be receptive to water with relatively low concentrations of Na^+^; thus, water with too much Na^+^ might be harmful, and an “appropriate Na^+^ concentration for absorbing during puddling” would exist. Actually, two *Papilio* males that sipped a 1.0 M NaCl solution in field experiments showed quite pathological behavior [5]. Moreover, male *P*. *xuthus* butterflies given a 10 mM Na^+^ solution produced larger spermatophores than those given pure water, 1 mM Na^+^, or 100 mM Na^+^ solutions [10], which was in accordance with the current results. The authors of [4] also wrote that “a total of 41 *Pieris napi* visited the 0.001 + 0.01 M Na^+^ concentration solutions, whereas 25 *P*. *napi* visited the 0.1 + 1 + 5 M Na^+^ concentration solutions”. *Papilio* butterflies have taste sensilla inside their proboscis [15, 5], and dose-response curves suggest that these sensilla are part of a taste-based safeguard system against the over-concentration of Na^+^ [5]. However, *Papilio* butterflies were even observed sipping from the dead bodies of crabs, birds, cats and rats as well as the dung and urine of mammals [16]. The concentrations of Na^+^ in these sources are generally higher than 10 mM; for example, the concentration in the blood of one of the article’s authors was approximately 140 mEq/L, and such observations are inconsistent with the current results. It is important to note that we only observed butterfly puddling at these sources after heavy rainfall or immediately following rain showers. Hence, the concentrations of Na^+^ in these materials might have been diluted to a level appropriate for *Papilio* butterflies, which attempted to absorb sufficient Na^+^ from these materials. As above, our previous results from the examination of Na^+^ concentrations in the water on which *Papilio* butterflies puddled in the field suggested that it was difficult for them to obtain sufficient Na^+^ from such water sources [5].

Three of 45 butterflies that were fed the PS absorbed K^+^, which might have been caused by organic substances in the PS. The molar ratio of Na^+^ to K^+^ and the changes in the Ca^2+^ and Mg^2+^ concentrations were not in accordance with the *Gluphisia* moth study [12]; these differences might be explained by the different test solutions used in [12] and the current study.

Finding NH_4_^+^ in the urine was an unexpected result because insects usually excrete N waste as uric acid. NH_4_^+^ in the urine might have been produced by post-collection chemical reactions because the samples were stored for up to eight months until analysis, although the samples were stored at -10°C. Because the muddy drops of puddling urine were not collected, we believe that contamination with uric acid and/or its related substances was minimal. NH_4_^+^ is the simplest nitrogenous waste substance, and if an animal obtained enough water, excreting such waste as NH_4_^+^ is reasonable.

We found that the concentrations of Na^+^ and K^+^ in Apiaceae and Aristolochiaceae plant species are similar to those of other Japanese Papilionini host plants (Table 5), therefore the differences in host plants cannot explain the differences in puddling frequency among Japanese *Papilio* species documented in our previous study [5, 16].

Certain butterflies including captured in the field refused to puddle with the test solutions. Our results (Table 6) indicate that the concentration of Na^+^ in flower nectar is generally greater than that in PS01. Similar results were also found in [13], who determined that the molar ratio of K^+^ to Na^+^ in the flower nectar was between 0.35 and 135.17, which nearly corresponds to our results (between 0.02 and 166.74). Thus, butterflies that had obtained enough Na^+^ from flower nectar in the field might not have needed to puddle on damp ground or our salt solutions. Therefore, the influence of K^+^ from flower nectar may be less important than the influence of K^+^ from host plant leaves. Our previous survey [5] showed that natural water that was puddled on by *Papilio* butterflies also contained more K^+^ than Na^+^. Thus, for adult butterflies, the Na^+^ concentration appears to be more important than the K^+^ to Na^+^ ratio.

All butterflies, male and female, excreted large amounts of K^+^ in the meconium compared with the other three ions; hence, the K^+^ excreted during puddling might represent what was retained in the bodies after pupation. The changes in Na^+^ and K^+^ concentrations in the hemolymph and the meconium after eclosion (0–5 hours) of *Pieris brassicae* were measured in [17]. The author also measured the Na^+^ and K^+^ changes in the hemolymph that resulted from diuresis after the eclosion of *Papilio demodocus*, *Acraea horta* and *Danaus chrysippus* [18]. Although the author did not distinguish between male and female samples, butterflies excreted more K^+^ than Na^+^, which was similar to the results of the current study.

Based on our results, another potential reason is that female butterflies excrete K^+^ not only in the meconium but also in the eggs. In our current study, the eggs (including the egg shell and a glue-like substance attached to each egg surface) of five *Papilio* species contained 2.6–5.8 μg of K^+^ (Table 9). We confirmed that all female butterflies of the Japanese *Papilio* species potentially lay more than 600 eggs in their lives; thus, all female butterflies need to have at least 1.5 mg of K^+^. This value is similar to the amount of K^+^ that was excreted by the male *Papilio* butterflies during puddling behavior (Tables 2–4). This requirement may explain why puddling is comparatively rare in female *Papilio* butterflies relative to male *Papilio* butterflies.

During the research, other issues were encountered; in the laboratory, certain butterflies absorbed solutions and produced urine, whereas others did not despite being reared on the same host plant leaves in the same way, emerging on the same day, and being housed and treated identically. Additionally, the differences in frequency of puddling behavior between males and females and young and old individuals (only young male butterflies are considered to puddle, although during field experiments in our previous research [5, 16], we confirmed that female and old male butterflies also puddle) and among closely related species still remain unexplained. Unexpected behaviors of Danaidae and Lycaenidae are issues for further study.

To resolve these issues, we must examine butterfly blood in addition to urine and analyze other metal ions, organic substances and nitrogenous compounds in addition to the four ions for which fluctuations are presented in the present article. In particular, the importance of Fe and Cu may differ between red-blooded animals and green-blooded animals because hemoglobin, which transports oxygen in red-blooded animals, contains Fe, whereas hemocyanin, which transports oxygen in green-blooded animals, contains Cu. Moreover, Zn and Mn are used to enhance the hardiness of insect larva mandibles [19] as well as functioning as enzyme cofactors. We are also interested in the fluctuations of these substances and have attempted to perform measurements using urine; however, we could not perform the measurements with our current system because the concentrations of these substances and ions in butterfly urine might be too low for accurate analysis. Because the amount of butterfly blood that we could obtain was too small for accurate analysis, continuous research on the fluctuation of these substances in butterfly blood will be more difficult.

In conclusion, the purpose of puddling in male *Papilio* butterflies is likely to adjust the concentrations of substances within their bodies. This exchange primarily consists of the excretion of K^+^ that the butterflies acquired from host plant leaves during the larval stage, and the absorption of Na^+^, of which butterflies obtain little from their typical diet. According to these results, the butterfly alimentary tract selectively absorbs or excretes each ion independently according to the concentration of ions in the absorbed water and in their hemolymph, such as in the Malpighian tubule, assuming the concentration of each substance in the absorbed water is adequate. We strongly recommend that those who rear *Papilio* butterflies in a laboratory or greenhouse prepare feeding solutions with adequate concentrations of metal ions not only to supply sufficient calories and Na^+^ but also to facilitate the excretion of excess K^+^.
